# Association of stress-sensitive mid-insula activity with alcohol drinking and negative affect-like behavior during abstinence in mice

**DOI:** 10.1016/j.neuropharm.2026.110859

**Published:** 2026-02-12

**Authors:** Benjamin M. Williams, Jincy R. Little, Samuel W. Centanni

**Affiliations:** Department of Translational Neuroscience, Wake Forest University School of Medicine, Winston-Salem, NC, USA

**Keywords:** Bed nucleus of the stria terminalis, Insular cortex, Stress, Alcohol drinking, Negative affect

## Abstract

Stress is central to many neuropsychiatric conditions, including alcohol use disorder (AUD). Stress influences alcohol initiation, escalation, progression to AUD, and relapse. Identifying stress-activated neurocircuits and individual variability in these responses is critical for developing new AUD treatment targets. This study investigates the relationship between adult stress response and AUD hyperkatifeia-a prolonged negative emotional state in protracted abstinence. In C57BL/6J mice, repeated restraint stress did not alter ethanol consumption but heightened aversive behavior during abstinence. We examined the mid-insula, a key network hub for emotional regulation and stress response, as a potential mechanism driving this effect. Mid-insula GCaMP activity was higher during active stress-coping behavior, and negatively correlated with ethanol consumption, and positively correlated with GCaMP activity during the novelty-suppressed feeding test in abstinence. Next, we assessed whether stress-induced activity in the stress-sensitive insula-BNST circuit is sufficient to alter ethanol drinking behavior and abstinence-induced avoidance behavior. Chemogenetically inhibiting mid-insula-BNST neurons during stress had sex-specific effects-reducing ethanol consumption in males and abstinence-induced aversive behavior in females. Clustering analysis revealed two distinct phenotypes-one characterized by high active coping during stress, low ethanol consumption, and low avoidance behavior in abstinence, and a second cluster with the opposite pattern. Insula-BNST inhibition during stress shifted female mice toward the former cluster, but had no impact on male cluster identity. Collectively, this study implicates the insula-BNST circuit as a key mediator of stress response, stress-induced drinking, and abstinence-related affective vulnerability, positioning this circuit as a potential biomarker and therapeutic target for hyperkatifeia.

## 1. Introduction

Stress is a pervasive and individualized experience that significantly contributes to the onset and exacerbation of numerous neuropsychiatric diseases, including mood, substance use, and anxiety disorders. Among these, problematic alcohol use stands out due to its profound impact on public health, with stress and emotional dysregulation intertwined with the initiation and continuation of alcohol use to alleviate stress, which, in some individuals, can lead to the progression to alcohol use disorder (AUD) (Sinha, 2022). AUD patients in abstinence often experience hyperkatifeia, or heightened sensitivity to emotional distress in drug or alcohol withdrawal (Koob et al., 2020). The inability to properly cope with stress is a key driver of relapse and cravings in many individuals (Sinha, 2024). Despite the clear link between stress and AUD, efforts to use stress response as a predictive marker have been hindered by its complexity and individual variability in stress response. Stressors can have drastically different short- and long-term impacts on individuals, and mental health conditions involve complex overlapping dysfunctional patterns that complicate pinpointing cause and effect.

Improving diagnosis and treatment strategies requires environmentally controlled preclinical studies to isolate the stress/alcohol relationship. Unfortunately, rodent models of stress-induced changes in AUD-related behavior have yielded ambiguous results. In rodents, stress can increase, decrease, or have no impact on alcohol drinking behavior, depending on the modality and duration of stress and alcohol exposure, as well as rodent strain and other environmental variables. Here, we deploy a model that relies less on overall stress-induced effects on alcohol drinking behavior and more on assessing behavior during a stressor and linking the stress response with individual differences in drinking patterns and alcohol-induced affective disturbances. Restraint or immobilization stress engages the HPA axis to increase glucocorticoid levels in rodents (Molina et al., 2023). One advantage of restraint is the bimodal behavioral response during stress; rodents exhibit either active or passive coping, with the former being pharmacologically validated to correlate with anxiety-like behavior (Grissom et al., 2008; Patel et al., 2005). Chronic drinking-forced abstinence (CDFA) is a mouse model that yields high voluntary alcohol consumption and affective disturbances in protracted abstinence (Holleran et al., 2016; Pang et al., 2013; Vranjkovic et al., 2018; Winters et al., 2021), with greater effects in female C57BL/6J mice (Centanni et al., 2019; Salazar and Centanni, 2024).

To bridge the gap between behavior and the neurobiology of these behavioral phenotypes, we can turn to neurocircuits the basic functional units that encode behavior. Neurocircuits could serve as a more effective indicator of AUD-related behavior like susceptibility to hyperkatifeia and stress-induced relapse, thus informing more individualized treatment strategies. The insula, a central hub in the brain’s salience network, regulates the switch between the default mode network and the executive control network (Ibrahim et al., 2019; Menon and Uddin, 2010) and conveys interoceptive cues through cortical and subcortical connections to predict outcomes and guide behavioral actions (Centanni et al., 2021; Namkung et al., 2017). Human imaging studies show increased insula activation in response to psychosocial stress, uncertain threats, and alcohol cues (Bach et al., 2024; Dager et al., 2014; Gorka et al., 2023; Myrick et al., 2004). In rodent models of stress and chronic alcohol exposure, we outlined the mid-insula, an insular subregion that integrates emotional response to stimuli, as a hyperactive area driving escape behavior during stress and increased alcohol abstinence-induced negative affect (Centanni et al., 2019; Luchsinger et al., 2021).

Despite the vast connectivity of the insula, its specific projections, such as those to the extended amygdala (Centanni et al., 2019; Gehrlach et al., 2020), have received comparatively little attention. The bed nucleus of the stria terminalis (BNST), a component of the extended amygdala that receives dense projections from the insula, integrates negative valence or affective states driven by cortical, subcortical, midbrain, and hindbrain inputs (Koob and Zorrilla, 2010; Reynolds and Zahm, 2005). We and others have demonstrated that binge (Marino et al., 2021) and chronic voluntary alcohol drinking (Centanni et al., 2019) drive increased glutamatergic signaling in the insula-BNST pathway. Using chemogenetics and slice electrophysiology, we linked these changes with increased negative affect-like behavior in protracted abstinence (Centanni et al., 2019; Taylor et al., 2024). A separate study shows increased insula-BNST activity at the onset of active stress-coping behavior during restraint stress, and chemogenetically increasing insula-BNST pathway activity during stress enhances post-stress avoidance behavior (Luchsinger et al., 2021).

To date, no study has examined how pre-alcohol stress response predicts drinking behavior and abstinence-induced negative affect-a critical gap, as early stress reactivity may serve as a predictive biomarker for AUD vulnerability and guide the development of targeted interventions. Building on prior work linking mid-insula and mid-insula-BNST pathway activity to heightened stress response (Luchsinger et al., 2021) and alcohol abstinence-induced negative affect-like behavior (Centanni et al., 2019), we test the hypothesis that mid-insula circuit activity predicts individual susceptibility to AUD-related behavior. Prior studies report more robust alcohol and stress effects in female mice, outlining this population as particularly important for studying stress-related AUD behavior. This increases the translational relevance as females exhibit higher rates of drinking to cope with stress, with growing evidence of faster progression to alcohol dependence and higher susceptibility to negative affect-related disorders (Agabio et al., 2016, 2017; Bangasser and Cuarenta, 2021; Grossman and Wood, 1993; Maciejewski et al., 2001). Accordingly, the present study uses female mice to dissect the neurobiological underpinnings of stress-induced vulnerability to AUD.

Using a longitudinal mouse model that combines restraint stress, continuous access two-bottle choice alcohol drinking, and protracted abstinence, we link pre-alcohol stress response with drinking patterns and negative affect in abstinence. We employed *in vivo* calcium imaging and circuit-specific chemogenetics to dissect the functional contributions of the insula→BNST pathway to AUD-related behavior. To assess the generalizability of these findings, this latter manipulation was evaluated in male and female mice, revealing distinct sex differences and sub-populations susceptible to hyperkatifeia in protracted alcohol abstinence. We propose a mechanistic framework linking stress-induced neuronal activity to AUD-related behaviors. By identifying the mid-insula-BNST pathway as a key modulator of stress-susceptibility, this study offers novel insights into the neurobiological underpinnings of AUD vulnerability, particularly abstinence-induced negative affect. These findings highlight the potential of this pathway for earlier intervention and personalized treatment strategies, shifting the focus from reactive to preventive approaches.

## 2. Methods and materials

See supplementary material for detailed methods.

Singly housed male and female C57BL/6J mice (n = 124; The Jackson Laboratory) were delivered at age seven weeks and acclimated for one week before experimentation. All procedures were conducted with the approval of the Institutional Animal Care and Use Committee at Wake Forest University and were within the guidelines set forth by the Care and Use of Mammals in Neuroscience and Behavioral Research (National Research Council, 2003).

**Fig. 1.**
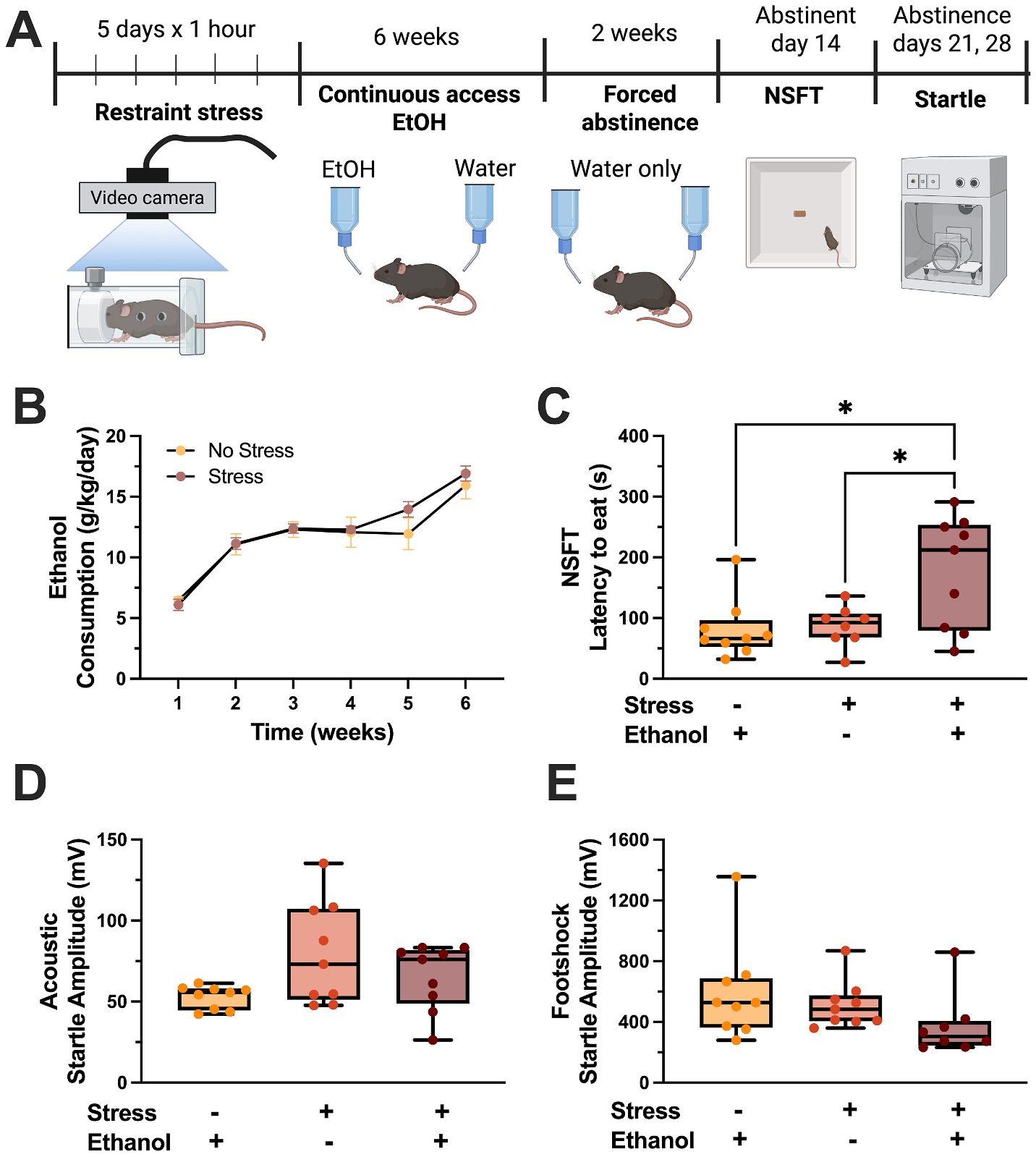
Repeated stress exposure enhanced negative affective phenotypes following 6 weeks of continuous ethanol drinking. (A) Experimental design. (B) Stress does not impact average weekly ethanol consumption. (C) Mice with prior stress and ethanol exposure show higher latency to eat the food during NSFT in abstinence, compared to stress and no ethanol controls. (D-E) Acoustic (D) and footshock (F) startle responses are similar between groups. (n = 8–9 mice/group, *p < 0.05).

Sub-chronic restraint stress and CDFA were conducted as previously described (Centanni et al., 2019; Holleran et al., 2016; Luchsinger et al., 2021) and in Fig. 1. All viruses were purchased from Addgene, used as received, and stereotaxically injected into the mid-insula and/or the dorsal BNST (300 nL). DREADD agonist 21 (C21, MilliporeSigma) was diluted in saline and administered (1 mg/kg, i.p.) 1h before stress. Mice were handled, and behavioral studies were performed as previously described (Holleran et al., 2016; Olsen and Winder, 2010; Pang et al., 2013).

All data were graphed and analyzed using RStudio, MATLAB, and GraphPad Prism 10, with a significance level of p < 0.05. For continuous outcomes (e.g., ethanol consumption) across multiple treatment/comparative groups, ANOVA was used, along with an appropriate method for multiple comparisons (e.g., Dunnett’s test) relevant to the hypotheses being tested. For simple two-group comparisons, Welch’s t-tests were used.

To identify patterns and number of clusters within our datasets, data were fit to a Gaussian Mixture Model (GMM), Principal Component Analysis, and Silhouette plots. Silhouette and hierarchical clustering plots were used to visualize the distribution and to determine the optimal number of clusters. Next, K-means clustering algorithms set to k = 2 were used to visualize multidimensional clustering. Cluster identity was determined using z-scored centroids to identify strength of each behavior in driving the cluster identities. Subsequent bootstrapping and downsampling analysis combined with Mahalanobis distance (De Maesschalck et al., 2000) and Fisher’s Exact test determined the robustness and stability of our model and clustering.

## 3. Results

### 3.1. Repeated restraint stress increases abstinence-induced negative affect-like behavior

To assess the impact of repeated stress on negative affect-like behavior during abstinence, female C57BL/6J mice underwent five days of restraint stress (1 h/day). Seventy-two hours after the last stressor, mice began the chronic ethanol drinking-forced abstinence model (CDFA) (Centanni et al., 2019), which consisted of 6 weeks of continuous access to ethanol followed by 2 weeks of forced abstinence (Fig. 1A). Restraint stress did not impact ethanol consumption (Fig. 1B). However, two weeks into abstinence, repeated stress exposure before drinking drove a significantly higher latency to eat during the novelty suppressed feeding test (NSFT) (one-way ANOVA, F(2,23) = 4.05, p = 0.031, Tukey’s post hoc stress effect p = 0.011, ethanol effect p = 0.021, Fig. 1C). To assess whether this effect generalizes across other behaviors evoking negative affect-like behavior, mice underwent acoustic and footshock startle tests. Neither assay resulted in an ethanol or stress effect (Fig. 1D and E), although acoustic startle showed a trend toward a treatment effect (one-way ANOVA, F(2,23) = 3.26, p = 0.059, Fig. 1D).

### 3.2. Mid-insular GCaMP activity during restraint stress is negatively correlated with ethanol consumption

**Fig. 2.**
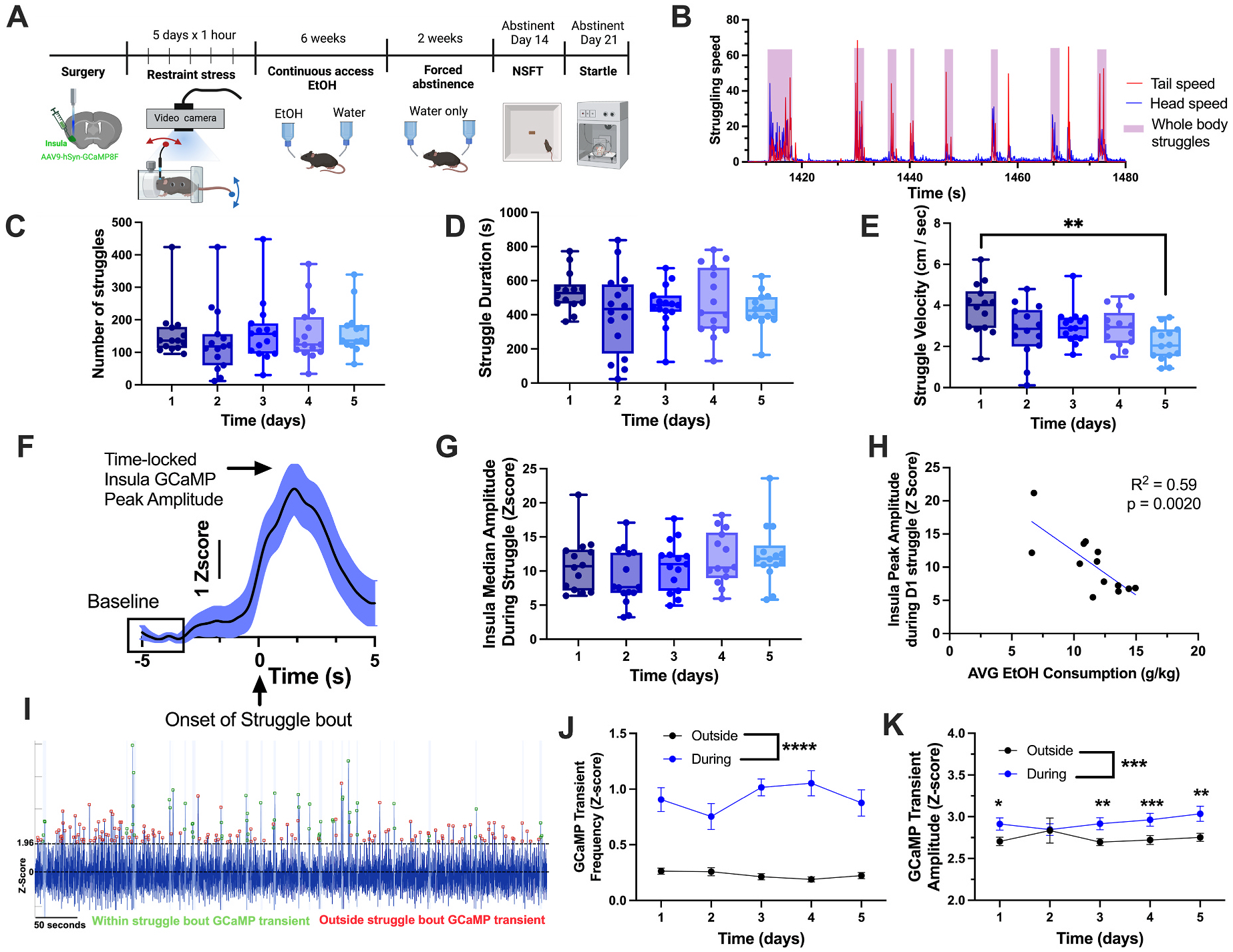
Mid-insular GCaMP response during restraint stress struggle bouts negatively correlates with ethanol drinking in female mice. (A) Experimental design for Figs. 2 and 3. (B) A representative trace illustrates the speed of tail, head, and whole-body struggle bouts during restraint stress, as measured with DeepLabCut and custom R code. (C) The number of struggling bouts remains unchanged over 5 days of restraint stress. (D) The total struggle duration throughout the 1 h of stress remains constant over time. (E) The average struggle velocity is significantly lower on day 5 compared to day 1. (F) Representative trace of insula GCaMP activity time locked with struggle bouts during restraint stress. Baseline determined using a z-score transformation of the median dF/F during a 2-s window before each bout onset (− 5 to − 3 s before onset) (G) Mid-insula GCaMP peak amplitudes, time-locked to struggle bouts, are similar across the 5 days of restraint stress. (H) Peak amplitude of insula GCaMP activity on day 1 of restraint stress negatively correlates with the average ethanol consumption during weeks 4–6. (I) Representative trace illustrating GCaMP transient spike frequency and amplitude during and outside of a struggle bout. Shaded area = struggle bout. Green boxes = transient within a struggle bout. Red box = transient outside of a struggle bout. The dashed lines represent the baseline calculated from the median dF/F across the entire recording (converted to Z = 0) and the threshold for spike transients (Z = 1.96). (J) Mid-insula GCaMP transient frequency is higher during struggle bouts compared to outside of struggle bouts across all days of stress. (K) Mid-insula GCaMP transients during a struggle bout have a higher amplitude than transients outside of struggle bouts across all stress days except for day 2. (n = 14 mice, **p < 0.01, ***p < 0.001).

We next sought to determine the neurobiological mechanism driving this distinct relationship between pre-CDFA stress exposure and NSFT behavior in abstinence. Given the unique role of the mid-insula in stress-coping behavior (Luchsinger et al., 2021) and ethanol abstinence-induced negative affect (Centanni et al., 2019), we investigated whether mid-insula activity during stress is an indicator of subsequent ethanol consumption. Mid-insula GCaMP activity in female mice was measured with *in vivo* fiber photometry during five days of 1-h restraint. Active escape attempts (struggle bouts), a validated measure of negative affect (Grissom et al., 2008; Luchsinger et al., 2021; Patel et al., 2005), were quantified using machine learning pose estimation (DeepLabCut) and custom R code (Fig. 2A and B). The struggle bout number and the total struggle duration did not change over consecutive days of stress (Fig. 2C and D). However, there was a significant effect of time in struggle velocity, driven by a decrease between the first and last days (Mixed Effects Model one-way ANOVA, p = 0.003, Tukey post-hoc test day1 vs day5: p = 0.001, Fig. 2E). Mid-insula GCaMP activity time locked to the onset of struggle bouts (Fig. 2F) revealed that peak GCaMP amplitude at the onset of a struggle bout was consistent over the five days of restraint stress (Armario et al., 2004; Grissom and Bhatnagar, 2009; Hennessy and Levine, 1977) (Fig. 2G). Mice underwent CDFA for 6 weeks following stress. There was a strong negative correlation between the average daily ethanol consumption over the last 3 weeks and the insula median GCaMP activity during the first day of stress (Simple Linear Regression R^2^ = 0.59, F(1,9) = 16.27, p = 0.0020, Fig. 2H). Interestingly, this negative correlation between drinking and mid-insula activity during stress was the strongest on the first day with declining significance after each subsequent day of stress (Pearson r correlation matrix, Fig. S1). To determine whether changes in insula activity are specific to struggling behavior, we compared insula spontaneous GCaMP transient events (Z-score >1.96) during and outside of struggling behavior (Fig. 2I). This Z threshold was chosen to identify statistical outliers 1.96 standard deviations above the mean baseline (p < 0.05). Spike frequency was significantly higher during a struggle bout compared to when the mouse was not engaging in struggling behavior, an effect consistent across all five stress days (Mixed Effects Model, F_(1,16_) = 105.7, p < 0.0001, Fig. 2J). In addition, the maximum peak amplitude of the GCaMP transients was higher during a struggle bout than outside (Mixed Effects Model, F(1,16) = 17.9, p = 0.0007, Fig. 2K). This suggests preferential recruitment of the mid-insula during struggle behavior.

### 3.3. Mid-insular GCaMP activity during restraint stress and negative affect-like behavior in abstinence

**Fig. 3.**
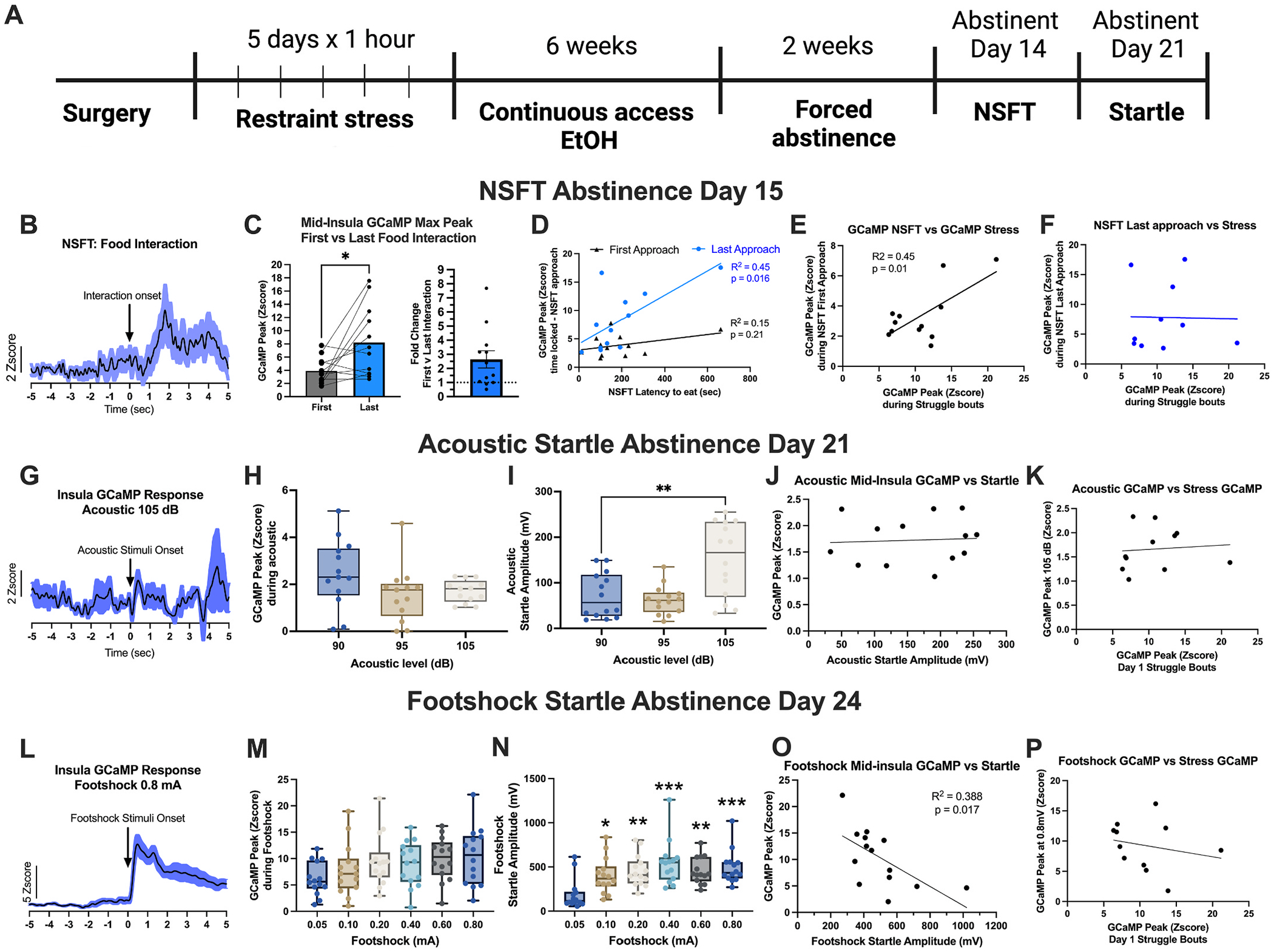
GCaMP activity in the mid-insula is active and correlates with negative affect-like behavior during NSFT and footshock startle, but not acoustic startle in female mice. (A) Timeline of experiments. (B) Mid-insula GCaMP activity, averaged across subjects, is time-locked to the last (consummatory) food interaction during NSFT. (C) Mid-insula GCaMP peak amplitude is greater during the last food interaction compared to the first food interaction. The left graph shows raw peak amplitudes for each mouse. The right graph shows data normalized to the fold change of GCaMP max peak between the first interaction and the last interaction. (D) Insula GCaMP activity time-locked to the last but not during the first food interaction during NSFT is positively correlated with the latency to eat during. (E-F) Mid-insula GCaMP activity time-locked to struggle bout onset during the first day of restraint stress is positively correlated with GCaMP activity time-locked to the first food interaction (E), but not the last food interaction during NSFT (F). (G) Representative traces of mid-insula GCaMP activity time-locked with 105 dB acoustic startle. (H) Mid-insula GCaMP peak amplitudes, time-locked to the acoustic stimulus, are similar across different decibel (dB) levels. (I) Peak startle response amplitudes (mV) to a 105 dB acoustic stimulus are higher than 90 dB. (J-K) Mid-insula GCaMP activity during acoustic startle does not correlate with either the (J) acoustic startle amplitude or the (K) mid-insula GCaMP activity time-locked to struggle bout onset during day 1 of stress. (L) A representative trace of mid-insula GCaMP activity time locked with 0.8 mA footshock. (M) Mid-insula GCaMP peak amplitudes during footshock startle are not different across shock levels. (N) Peak startle response amplitude to a footshock stimulus increases with higher shock levels. (O) Peak mid-insula GCaMP activity amplitude during a footshock stimulus negatively correlates with the footshock startle amplitude. (P) Peak mid-insula GCaMP activity amplitude during restraint stress struggle bouts does not correlate with the mid-insula GCaMP peak amplitude at the onset of footshock stimulus (n = 12– 14 mice/test; *p < 0.05, **p < 0.01, ***p < 0.001).

After stress and ethanol drinking, we examined the relationship between pre-CDFA stress-activated mid-insula activity and negative affect-like behavior during abstinence in the same cohort, as shown in Fig. 2. Two weeks into abstinence, mid-insula GCaMP activity was measured during NSFT, time-locked to each interaction with the food pellet. An approach was defined as when the mouse moved within 2 cm of the food pellet. The peak height of the GCaMP signal was significantly higher during the last (i.e., consummatory) food interaction compared to the first (paired *t*-test, t(12) = 2.65, p = 0.023, Fig. 3B and C). The peak GCaMP amplitude time-locked with the last, but not the first food interaction, positively correlated with the latency to eat during NSFT (Simple Linear Regression, last: R^2^ = 0.45, p = 0.017; first: R^2^ = 0.15, p = 0.21, Fig. 3D). In addition, a positive correlation emerged between stress-induced mid-insula GCaMP peak amplitude from the first day of stress exposure and GCaMP peak amplitude during the first food interaction during NSFT 8 weeks later (R^2^ = 0.45, F(1,10) = 8.34, p = 0.017, Fig. 3E). Stress-induced peak GCaMP amplitude did not correlate with the last food interaction on NSFT, immediately prior to the food consumption (Fig. 3F). The peak amplitude during stress days 2–5 showed a gradually weakening positive correlation over time with the first food interaction and no correlation with the last food interaction (Pearson r correlation matrix, Fig. S2).

In contrast, mid-insula activity was not recruited during the acoustic startle test on abstinence day 21 (Fig. 3G and H), despite increasing decibels evoking a stronger startle response (repeated-measures (RM) one-way ANOVA, F(2,39) = 2.79, p = 0.01, Tukey’s post-hoc test 90 dB vs 105 dB: p = 0.0026, Fig. 3I). The behavioral response to the highest decibel (105 dB) was not correlated with mid-insula GCaMP activity time locked to stimulus presentation (Fig. 3J). Pre-CDFA restraint stress insula GCaMP activity did not correlate with GCaMP activity during the acoustic startle response (Fig. 3K).

During the footshock startle test on abstinence day 24, all shock levels recruited robust mid-insula GCaMP activity (Fig. 3L and M). Startle amplitude after a footshock was higher in the five highest intensities compared to the lowest intensity (0.05 mA) (RM one-way ANOVA, F(5,83) = 6.34, p < 0.0001, Dunnett’s multiple comparisons: 0.05 versus 0.1: p = 0.01, 0.05 versus 0.2: p = 0.0016, 0.05 versus 0.4: p < 0.0001, 0.05 versus 0.6: p = 0.0017, 0.05 versus 0.8: p = 0.0002, Fig. 3N). Comparing each mouse’s peak insula GCaMP amplitude during the highest shock intensity (0.8 mA) with the behavioral response revealed a negative correlation between GCaMP and startle response (R^2^ = 0.388, F(1,12) = 7.61, p = 0.017, Fig. 3O), indicating that mice with higher insula activity during stimulus exposure exhibited a lower behavioral response to the shock stimulus. In contrast, no correlation was observed between pre-CDFA stress GCaMP activity and GCaMP activity during footshock startle (Fig. 3P). We performed a comprehensive correlation analysis comparing insula GCaMP peak amplitude time-locked to aversive behavioral events (e.g., struggle bout onset, food interaction, stimulus presentation). Overall, no notable correlations emerged beyond the day 1 stress response and NSFT last food interaction described above (Fig. S2).

### 3.4. The mid-insula to BNST pathway encodes susceptibility to negative affect-like behavior in protracted abstinence

**Fig. 4.**
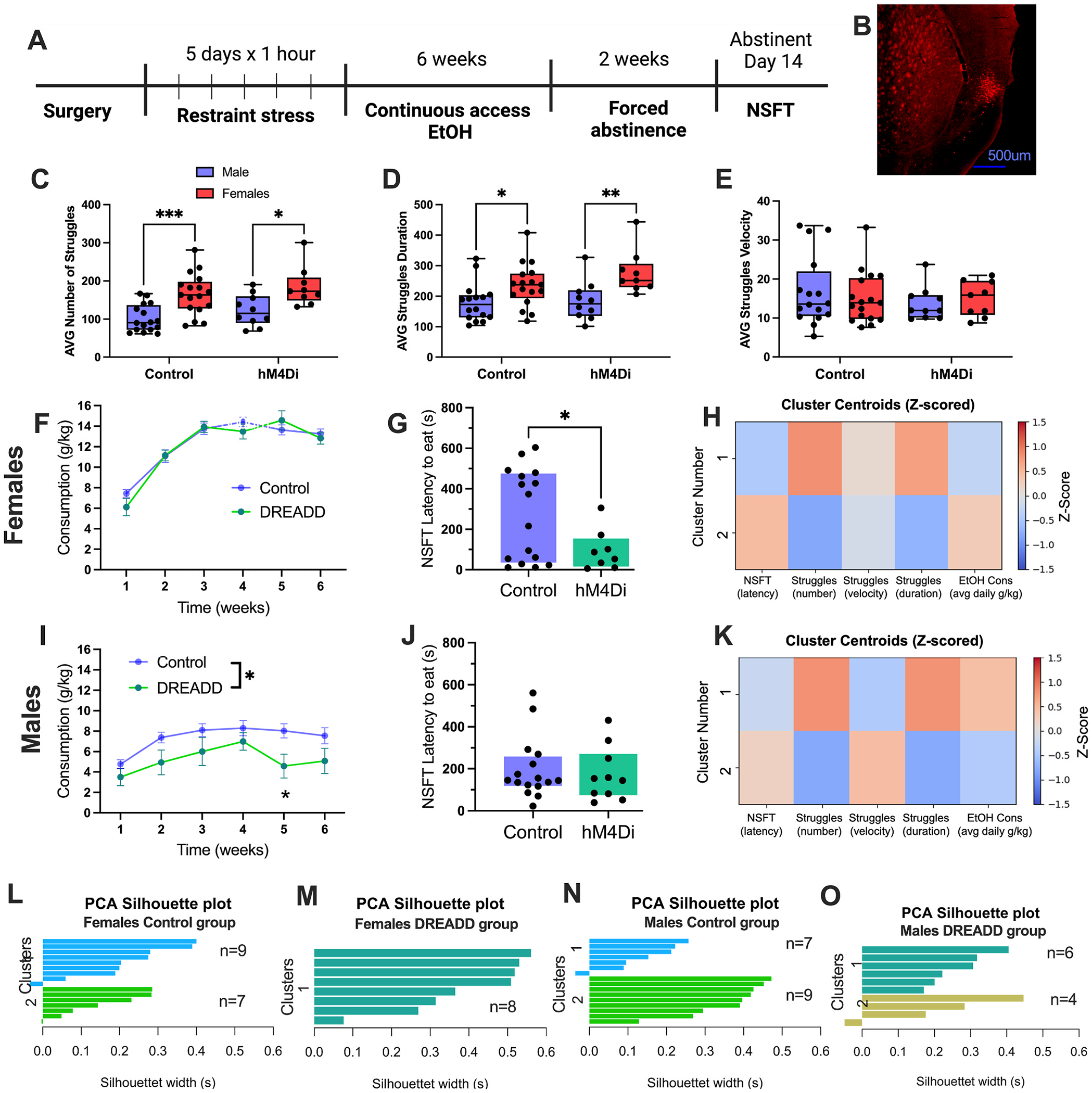
Chemogenetic inhibition of the insula-BNST pathway blocks the emergence of a stress-vulnerable sub-population. (A) Experimental design. (B) Representative image of hM4Di expression in the mid-insula. (C) The average number of struggle bouts across five days of repeated restraint stress is greater in females compared to males, but there is no difference between the control (mCherry-C21 or hM4Di-saline) and hM4Di (hM4Di-C21) groups. (D) The total struggle duration is greater in females compared to males in the control and hM4Di DREADD groups. (E) The average struggle velocity is not different between males, females, or viral groups. (F-G) Female data. F) Insula-BNST hM4Di activation during stress has no impact on ethanol consumption. (G) Latency to eat during NSFT after two weeks of abstinence was significantly higher in control mice compared to hM4Di mice. H) Heatmap displaying the Z-scored centroids for the two macro-clusters. Each row represents a cluster centroid, defined as the mean vector of all mice assigned to that phenotype across the behavioral dimensions. Positive values (red) and negative values (blue) represent behavioral measures above and below the average for the group, respectively. Cluster 1 contains high struggle duration and number, and low NSFT latency and ethanol consumption. Cluster 2 is driven by the opposite pattern. (I-K) Male data I) Insula-BNST hM4Di activation during stress drove lower ethanol consumption compared to controls. J) Latency to eat during NSFT in male mice is not affected by prior inhibition of the insula-BNST pathway during stress. K) Heatmap displaying Z-scored centroids for the two macro-clusters. Male mice are driven by struggle number and duration, and to a lesser degree NSFT latency in abstinence and ethanol consumption. (L-O) A silhouette clustering analysis including all the above behavioral data, color-coded by cluster identity described above. (L, N) Silhouette plots displayed two distinct clusters across all variables in both male and female control groups. (M) A silhouette plot of female hM4Di DREADD mice indicates a shift in representation from cluster 2 to 1. (O) A silhouette plot of male hM4Di DREADD mice. hM4Di mice showed similar cluster distribution compared to controls. (n = 8–17 mice/group *p < 0.05, ***p < 0.001).

To gain a circuit-level understanding of the mid-insula’s role in the stress-abstinence relationship and stress susceptibility, we examined the mid-insula to bed nucleus of the stria terminalis (BNST), a unidirectional circuit with enhanced activity during stress and in alcohol-related behavior. Using a pathway-specific inhibitory chemogenetic strategy, we inhibited the insula-BNST circuit during each stress exposure. We tested the hypothesis that decreasing insula-BNST activity reduces stress saliency and prevents heightened negative affect in individuals in abstinence. Although the primary focus thus far has been female mice, for this study, we conducted parallel cohorts of male and female mice to examine whether this circuit underlies established sex differences in stress and AUD-related behavior, an area that has not been studied. Mice received bilateral retrograde Cre-recombinase injections into the BNST and Cre-dependent Gi-DREADD (hM4Di) into the mid-insula (Fig. 4A). Stress control groups had either C21 injections and no hM4Di virus or vehicle injections with the hM4Di virus to assess independent effects of C21 and hM4Di. Chemogenetic inhibition of insula→BNST neurons occurred with C21 (1 mg/kg i.p.) 1 h before each stress exposure. Interestingly, female mice consistently engaged in more struggle bouts than male mice (two-way ANOVA, sex: F(1,49) = 23.43, p < 0.0001, Tukey’s post hoc, controls: p = 0.0003, hM4Di: p = 0.010, Fig. 4C), but there was no overall effect of DREADD (two-way ANOVA, DREADD: F(1,49) = 2.63, p = 0.11). A similar effect was observed with struggle bout duration (two-way ANOVA, DREADD: F(1,49) = 1.69, p = 0.19; sex: F(1,49) = 15.7, p = 0.0002; Tukey’s post hoc, controls: p = 0.028, hM4Di: p = 0.0076 Fig. 4D). There were no differences in struggle velocity (i.e., intensity, Fig. 4E). Thus, while sex differences emerged, chemogenetically decreasing insula→BNST activity did not affect struggling behavior (Fig. 4C–E).

Following stress exposure, mice were subjected to six weeks of continuous access to 10% ethanol with two weeks of forced abstinence. Insula→BNST hM4Di during stress resulted in lower ethanol consumption in male (two-way ANOVA, F(1,24) = 6.63, p = 0.016; Fig. 4I), but not female mice (Fig. 4F). There was an effect of time in both male and female groups (two-way ANOVA, females: F(2,63) = 84.10, p < 0.0001; Fig. 4F; males: F(3,83) = 8.56, p < 0.0001, Fig. 4I), suggesting escalation in drinking. Ethanol preference was also significantly reduced in male hM4Di mice, but not in female mice (two-way ANOVA, F(1,24) = 6.63, p = 0.016, Fig. S3A–B).

Following two weeks of abstinence, mice were tested in the NSFT to measure negative affect-like behavior. Chemogenetically inhibiting the insula-BNST pathway during stress resulted in lower latency to eat (Welch’s *t*-test, t(19.44) = 2.564, p = 0.0188, Fig. 4G). This effect was not present in male mice (Fig. 4J). Food consumption after NSFT was tested for 10 min in the mouse’s home cage. There was no effect of hM4Di on post-NSFT food consumption in males or females (Fig. S3C–D). However, there was a negative correlation between EtOH consumption and latency to eat during NSFT in the male control group, and a trending correlation in the hM4Di group (Control: R^2^ = 0.50, p = 0.0015; hM4Di: R^2^ = 0.37, p = 0.061; Fig. S3E), with no correlations present in female mice (Fig. S3F). In addition, there was a positive correlation between weight loss during the pre-NSFT food deprivation period and latency to eat on NSFT in control male mice, with a trending correlation in hM4Di mice (Control: R^2^ = 0.56, p = 0.004; hM4Di: R^2^ = 0.38, p = 0.078; Fig. S3G). This effect was similarly absent in female mice (Fig. S3H).

We hypothesized that a subset of female stress + CDFA mice were susceptible to stress, in turn driving the effect. To explore this, we fitted the control stress + CDFA data to a Gaussian Mixture Model (GMM), which identified two Gaussian distributions (Component 1: mean = 61.3 ± 63.5 s; Component 2: mean = 477.5 ± 73.8 s, Fig. S4A), supporting bimodality within the dataset. A similar analysis was performed on female hM4DI and both male groups (Fig. S4B–D). While the control male mice showed a potential bimodal distribution, it was less pronounced than that of female control mice. The female hM4Di groups exhibited no bimodal distribution, whereas the male hM4Di mice showed a weaker, but prominent bimodal distribution.

Cluster occupancy analysis revealed that two distinct macro-phenotypes characterized the Control population. In female control mice, the two clusters are strongly driven by struggle number and duration, and to a lesser extent by NSFT latency and ethanol consumption (Fig. 4H). Insula-BNST pathway inhibition during stress led to a shift away from cluster 2 (low struggle number/duration, high NSFT latency, high consumption) toward cluster 1. Although this did not reach categorical significance (Fisher’s Exact Test, p = 0.22), the numerical shift is noteworthy (Controls: Cluster 1 = 8 mice, Cluster 2 = 8 mice, hM4Di: Cluster 1 = 8 mice, Cluster 2 = 0 mice). Multivariate analysis confirmed that these phenotypes remain highly distinct, with a Mahalanobis distance of 3.4, a measure of covariance between groups that uses distance from the center of a multivariate distribution (De Maesschalck et al., 2000). In male mice, we similarly observed 2 clusters (Fisher’s Exact Test, p = 0.71; Mahalanobis distance = 3.9), driven by struggle behavior (Fig. 4K), but hM4Di had no impact on cluster distribution (Controls: Cluster 1 = 9 mice, Cluster 2 = 7 mice; hM4Di: Cluster 1 = 6, Cluster 2 = 4 mice). This suggests a more substantial influence of the insula-BNST pathway on cluster fidelity, and thus behavioral state influence, on females in this study.

To support the observed clustering effects, computational principal component analysis (PCA) was performed to identify and visualize the optimal number of clusters. The PCA included total struggle bouts, struggle velocity, struggle duration, EtOH consumption, and NSFT latency. Hierarchical cluster plots and silhouette width analysis were used to determine the optimal number of clusters within the dataset (Fig. 4J–M). Both female and male stress control mice fit into two distinct clusters, with average silhouette widths of 0.19 and 0.35 for females and males, respectively (where more positive values indicate a stronger fit within that cluster and a lower average distance to the other points within the cluster; Fig. 4J–L). However, only one cluster emerged within the male and female DREADD groups that received C21 during stress, with higher silhouette scores of 0.35 and 0.24 for females and males, respectively (Fig. 4O–Q). Hierarchical cluster dendrograms confirmed these observations (Fig. S4E–H). Based on these results, we implemented the factoextra clustering algorithm to visualize the data, setting the number of clusters to two (Fig. S4I–L). There were two distinct clusters in the control groups (Fig. S4I and K), but only one distinct cluster in the female DREADD group (Fig. S4J). Notably, this algorithm identified a second cluster in the male DREADD data (Fig. S4L), suggesting that the insula-BNST pathway may be more sensitive in females than in males.

Next, we tested the robustness of our model by evaluating potential bias from unequal group sizes. To assess distributional modality and phenotypic separation, we ran a sex-stratified pooled (control + hM4Di) sensitivity analysis using stratified bootstrapping (preserving the ratios) and balanced downsampling (matching controls to hM4Di). For females (Supplemental Table 1), k-means cluster assignments were stable, yielding a strong Mahalanobis distance (3.4) between phenotypes. Under balanced downsampling, the pooled covariance trace, a measure of total behavioral variance, exhibited high stability (CV = 2.63%), confirming the PCA loadings and internal validity indices remained consistent with the original results (median ARI, Silhouette). GMM model selection by Bayesian Information Criterion (BIC) consistently favored the same number of components. We observed very similar stability metrics in males (CV = 2.86%; Supplemental Table 2). These analyses demonstrate that our cluster definitions and the resulting phenotypic structure are robust to the unequal group sizes and are not driven by unequal sample sizes.

Lastly, to determine whether CDFA is required to produce a bimodal population distribution of stress responsive mice, alcohol-naive mice underwent repeated restraint stress, and two weeks later, testing on NSFT was conducted (Fig. S5A). Repeated restraint stress alone did not produce distinct clusters based on NSFT latency. Additionally, chemogenetic inhibition of the insula-BNST pathway during stress did not affect NSFT latency in female mice (Fig. S5B). This suggests that ethanol exposure is necessary to reveal stress-resilient and susceptible subpopulations.

## 4. Discussion

Stress exposure recruits highly individualized behavioral and neural responses. Isolating the unique differences in genetically and environmentally controlled mouse models allows for direct comparisons between a basal stress response and a constant period of heightened negative emotionality, often referred to as hyperkatifeia in SUD/AUD abstinence. We demonstrate a distinct role for the mid-insula, and specifically the mid-insula-BNST pathway, in encoding stress response and susceptibility to chronic alcohol drinking-induced hyperkatifeia. *In vivo* recordings in the mid-insula during acute and repeated restraint stress revealed a robust increase in GCaMP activity, primarily restricted to periods when mice engaged in struggling behavior. This signal correlated with future drinking behavior and insula activity during tNSFT in protracted abstinence. We identified the emergence of two distinct subsets of stress-exposed mice-one population exhibiting heightened avoidance behavior in protracted abstinence, while the other did not. Chemogenetically inhibiting the insula-BNST pathway during stress disrupted the development of stress-induced susceptible clusters, resulting in behavioral effects that persisted into protracted abstinence. Together, we characterized a model for stress and chronic alcohol exposure that uncovers the relationship between stress response and negative affect-like behavior during abstinence in female mice, an understudied population in the treatment of AUD. Embracing the individual variability in stress response and the differential recruitment of stress-sensitive neurocircuits has the potential to impact risk assessment, diagnosis, and treatment for stress-related and AUD.

### 4.1. Mid-insula GCaMP activity as a neural correlate for stress response behavior

The primary goal was to elicit a quantifiable behavioral response to stress and compare this response to the negative affective state induced by abstinence, by directly characterizing the active neurocircuitry and behavioral response during stress and in abstinence. Five days of 1-h restraint stress was chosen to produce a sustained stress response while limiting habituation that occurs with repeated exposure to homotypic stressors (Grissom et al., 2008; Luchsinger et al., 2021; Patel et al., 2005). This timeline had little impact on active coping behavior during stress, although struggle velocity, a measure of intensity, decreased on the fifth day, likely reflecting the beginning of stress desensitization. This finding is consistent with other studies (Grissom et al., 2008; Patel et al., 2005), despite differences in quantification methods and stress duration (30 min vs. 1 h), demonstrating the robustness of this model for inducing stress.

We successfully replicated our previous findings that the mid-insula is recruited at the onset of struggle bouts (Luchsinger et al., 2021). Here, we conducted a detailed analysis to determine whether this relationship persists outside of struggling bouts (i.e., passive coping states). Mid-insula spike transients during and outside of struggle bouts were assessed using a whole-trace z-score analysis method (Martianova et al., 2019; Zhang et al., 2010) modified for our data (Wallace et al., 2025). This revealed higher transient frequency and peak amplitude during struggle bouts relative to outside. Thus, the mid-insula is dynamically recruited during active coping behavior, perhaps to encode the salience of stress or the affective state. Conversely, decreased mid-insula transient frequency and amplitude during passive coping may reflect behavioral inhibition or learned helplessness. Of note, this effect did not diminish with repeated exposure to the same stressor, highlighting a role for the insula in continual integration of stress salience.

### 4.2. Restraint stress and chronic ethanol drinking drive heightened avoidance behavior but not startle response in protracted abstinence

Repeated restraint stress before CDFA resulted in behavior modality-specific effects. Female mice in protracted abstinence exhibited higher latency to eat during NSFT. While we predicted that this pattern would generalize to other negative-affect-like behavioral tests, the startle response elicited by acoustic or footshock stimuli did not yield group differences (Fig. 1). Several avenues can be explored to explain this seeming discrepancy. First, while other studies have shown abstinence-induced changes in negative affect-like behavior outwards of 35 days into abstinence in mice (Holleran et al., 2016), NSFT was the first behavioral test in abstinence, potentially evoking a unique response. In addition, NSFT was the only test conducted in a food-restricted state, which could recruit the gustatory cortex within the insula. Weight loss during the NSFT food deprivation was correlated with higher latency to eat on NSFT exclusively in male mice, where less bodyweight loss was associated with higher latency (Fig. S3). This was similar to a negative correlation between ethanol consumption and NSFT latency in males. This suggests that in males, NSFT may be less driven by anxiety-like behavior and more so by consummatory behavior and satiety.

Another notable difference between NSFT and startle tests is the conflict and cognitive processing involved in NSFT, whereas startle tests assess the behavioral response to a sudden, unexpected aversive stimulus. We predicted the startle tests would model a re-exposure to a mild aversive stressor, producing a heightened response in mice with prior stress experience. Future studies should examine re-exposure to the same stress in abstinence to test this idea. In addition, testing different stress exposures before drinking and the same or other aversive stimuli in abstinence will address the specificity of the stress and abstinence-induced negative affect relationship.

Lastly, a startle response reflects an active coping or escape behavior, akin to the struggle bouts quantified during restraint stress. The acoustic startle data for stress-only mice (Fig. 1D and Fig. 3H–J) were notably more variable than those for ethanol-only mice, which may reflect stress-induced variability in passive versus active coping strategies. The shock startle drove a trend towards a lower startle response in the stress + CDFA mice, which could be interpreted as an increase in freezing behavior. Testing these trends in a full fear conditioning paradigm will resolve the differences in startle response tests and NSFT. This study focused on the synergistic effect of stress and ethanol relative to stress and ethanol alone to model a typical human behavioral phenotype. However, given the prevalence of stress and alcohol use in our society, additional comparisons that include an unstressed, ethanol-naïve group, for example, will provide valuable insight into these findings.

### 4.3. Restraint stress as a sex-specific behavioral indicator of hyperkatifeia

Notable basal sex differences were observed in the stress response. Male mice were less susceptible to the effects of restraint stress, as evidenced by fewer overall struggle bouts and struggle duration compared to female mice (Fig. 4C and D). Interestingly, there were no sex differences in struggle intensity, suggesting distinct mechanisms for passive/active coping choice and active coping motor execution. The clustering of susceptible and resilient mice based on NSFT latency was stronger and more evenly distributed in female mice as well (Fig. 4H and I). Additionally, the CDFA model generally yields lower ethanol consumption and less abstinence-induced negative affect on NSFT in males, highlighting sex differences in our model. Overall, this model is stronger at detecting individual differences in female mice. This emphasizes the importance of developing and utilizing stress and alcohol models that capture resilient and susceptible female phenotypes.

In stress-exposed mice, higher negative affect-like behavior during NSFT in abstinence appeared to be driven by a subset of mice, mice susceptible and resilient to heightened negative affect in abstinence (Fig. 4E). As noted above, this effect did not generalize to startle tests. Thus, we pursued additional analyses and testing focused on NSFT. Importantly, tests for hyponeophagia, like NSFT, can yield bimodal behavior distributions in some models (Stedenfeld et al., 2011), a valuable feature for evaluating pharmacological treatments with high non-responder rates, like many antidepressants. In female mice, struggling behavior was not predictive of stress susceptibility (Figure S2). Instead, the underlying neural response to the stress was a better predictor of negative affect-like behavior in abstinence (Fig. 3).

### 4.4. Repeated stress and continuous access ethanol drinking in C57BL/6J mice

Our approach provides insight into how repeated restraint stress alters drinking behavior in a continuous access model. While the influence of stress on drinking behavior in mice has been widely studied, no consensus has emerged regarding its effects. Several factors influence variability in outcomes, including mouse strain, length and modality of stress, alcohol exposure model, timing of stress relative to alcohol exposure, and sex (Becker et al., 2011). In our study, repeated restraint stress (five days of 1-h sessions) did not impact drinking patterns in female or male mice (Figs. 1B and 4D). The absence of a definitive stress effect on ethanol drinking could relate to the continuous access model, the repeated stress duration, desensitization to the homotypic stressor, or a combination of these factors. The effects of stress on subsequent drinking patterns in female mice has not been widely tested. Human studies suggest females use alcohol to cope with stress at a higher rate than males (Fox and Sinha, 2009; Guinle and Sinha, 2020). Alternative models utilizing limited access schedules or testing in ethanol-dependent mice may be better suited to characterize the binge drinking patterns associated with stress relief or negative reinforcement (Cho et al., 2019). Moreover, directly comparing difference stress and alcohol exposure modulaties will help uncover the nuanced effects of stress on drinking patterns in male and female mice.

### 4.5. The mid-insula plays distinct, yet overlapping, roles in stress response and abstinence-induced negative affect-like behavior

Mid-insula GCaMP activity during the first food interaction on NSFT was significantly lower than during the last consummatory food interaction. However, when comparing peak GCaMP amplitude during a struggle bout during the first stress day with peak mid-insula GCaMP during the first or last food interaction, the first approach positively correlated with stress, but not the last approach, uncovering a specific relationship between mid-insula response to stress before drinking and during a negative-affect evoking environment in abstinence. We posit that during initial restraint stress exposure, a failed escape attempt signals heightened anxiety. However, after repeated exposures, the consequence of a failed escape becomes minimal, as the mouse learns that the stressor will subside regardless of the escape’s success. In NSFT, heightened insula activity during the consummatory interaction may reflect increased sensitivity with each interaction, culminating in food consumption and a more vulnerable state. The increased insula GCaMP activity during the consummatory bout contrasts with observations in the dBNST, a downstream target of the mid-insula. dBNST GCaMP activity increased at the onset of all food interactions (Jaramillo et al., 2020), suggesting the mid-insula tips the scale in overcoming hyponeophagia to drive consumption, potentially by modulating interoceptive hunger or shifting food valence through heightened activity relayed to the BNST. Notably, the mid-insula GCaMP signal was time-locked to the food interaction (mouse’s nose coming within 2 cm of the food), rather than the initiation of consumption. The insula is heavily integrated with the gustatory cortex. Therefore, consumption could engage these independent pathways. We aimed to separate the role of the insula in taste perception, conflict, and ultimately, the choice to consume the food. While the GCaMP study exhibited insufficient power (1-β) to conduct a complete clustering analysis (as in Fig. 4), this effect appears to be driven by a subset of mice that exhibited a significant (>2-fold) increase in mid-insula GCaMP peak amplitude activity during the first versus last food interaction in NSFT (Fig. 3C). This is consistent with the susceptible populations identified in Fig. 4, suggesting that the mid-insula is a neural correlate of stress susceptibility and resilience.

### 4.6. The mid-insula to BNST pathway differentially encodes stress response and alcohol-related behavior in male and female mice

Given the extensive interconnectivity and functional diversity of insula neurons, different populations may be recruited during stress and NSFT in abstinence. For example, a recent study outlines distinct firing patterns in subsets of anterior insula neurons during drinking (Starski et al., 2024). We targeted the mid-insula to BNST projection, given its role in negative affect-like behavior following acute stress exposure and during protracted alcohol abstinence (Centanni et al., 2019; Luchsinger et al., 2021). Moreover, the BNST is a highly sexually dimorphic brain area (Allen and Gorski, 1990), therefore we directly compared the effects of circuit manipulation during stress in male and female mice. Female mice exhibited more struggle bouts and a longer overall struggle time during restraint stress than males (Fig. 4C and D). Surprisingly, chemogenetic inhibition of the insula→BNST neurons during stress did not impact stress behaviors in either sex. However, in the abstinence condition, an effect emerged in female mice, with significantly lower latency to eat on NSFT in hM4Di mice. Our previous work (Centanni et al., 2019) identified that chemogenetic inhibition (hM4Di) of the insula-BNST pathway reduced latency to eat during NSFT but not in control water-drinking mice. Pathway inhibition before the forced swim test in ethanol (no stress) mice also reduced immobility time. Taken together with our work here, the insula-BNST pathway may encode the salience of a stressor, independent of prior stress experience. Thus, the insula-BNST pathway is essential for regulating negative affect behavior specifically during abstinence from alcohol, although a no-stress + no-ethanol and no-stress +ethanol group would be needed to firmly draw this conclusion.

Upon deeper statistical analysis that incorporated stress response, drinking, and NSFT behavior, two clusters of mice emerged within the control groups. These clusters were driven by struggling behavior during stress, and with NSFT latency and average ethanol consumption contributing lesser roles. Pathway inhibition during stress reduced the number of clusters to one in females, shifting mice toward cluster 1, which is defined by lower NSFT latency, lower ethanol consumption. This establishes the insula-BNST circuit as a critical mediator of future behavioral responses during heightened negative emotional states, such as protracted abstinence. Interestingly, cluster 1 is also associated with higher struggling behavior during stress. This could reflect a unique role of the insula-BNST pathway in the choice of coping strategies (active versus passive). In male mice, two similar clusters emerged, however hM4Di did not affect the distribution of mice within these two clusters. This suggests a more substantial influence of the insula-BNST pathway on cluster fidelity in female mice, reinforcing the strength of this model for delineating unique sex differences in stress and alcohol use disorder-related behavior. Moreover, this cluster phenotype may underlie a unique stress susceptible and resilient phenotype, something that can be examined directly in future studies.

Manipulating the insula→BNST pathway during stress affected drinking in male, but not female mice, with overall lower ethanol consumption and preference, and male hM4Di mice relative to control stress mice. This suggests a more pronounced link between the insula→BNST pathway activity during stress and the pharmacological effects of alcohol and consumption patterns in males. This could reflect a shift in the hedonic value of alcohol, with its aversive properties outweighing the subjective rewarding impact, a phenomenon more pronounced in continuous access compared to limited access models. Furthermore, average daily ethanol consumption was negatively correlated with NSFT latency in males, and insula-BNST hM4Di during stress weakened this correlation, suggesting higher alcohol consumption may increase resiliency to negative affect in abstinence. In contrast, female insula→BNST circuit activity during stress may be more related to future negative affect-like behavior, suggesting other factors like timing of ethanol exposure are more indicative of negative affect. Collectively, the data indicate that insula→BNST pathway activity is a better predictor of subsequent hyperkatifeia in females and consummatory patterns in males. Future studies should determine whether inhibiting insula-BNST projections during stress alters peripheral endocrine responses, such as corticosterone release. Establishing this link could clarify how circuit-level modulation contributes to sex-specific behavioral outcomes and individual differences in stress susceptibility. Additionally, while the estrous cycle was not monitored in the present study, hormonal fluctuations can influence stress-related behaviors (Albert and Newhouse, 2019; Ranabir and Reetu, 2011) and may interact with the insula-BNST circuit activity. Measuring estrous cycle-induced hormonal fluctuations, along with corticosterone and behavioral endpoints, will be necessary to understand the mechanisms underlying the bimodal distribution of behavioral NSFT responses in females.

### 4.7. Conclusion

We uncovered a distinct link between stress response and negative affect in protracted alcohol abstinence, revealing unique sex-specific stress effects that define female mice as either susceptible or resilient to abstinence-induced hyperkatifeia. We outline a mechanistic link from the mid-insula, via its projection to the BNST, to stress susceptibility. Together, this work underscores the critical need for sex-specific approaches to understanding stress responses and coping strategies. By dissecting how the mid-insula→BNST circuit is recruited by stress, we can pave the way for more precise diagnostics and transformative, individualized treatment strategies for AUD.

## Supplementary Material

1

## Data Availability

All data and analysis tools will be made available upon request.

## Appendix A. Supplementary data

Supplementary data to this article can be found online at https://doi.org/10.1016/j.neuropharm.2026.110859.
