# Supplementary material for: Association of stress-sensitive mid-insula activity with alcohol drinking and negative affect-like behavior during abstinence in mice
Source: Neuropharmacology. Author manuscript; Available in PMC 2026 Jun 5. (PMC13238140; doi:10.1016/j.neuropharm.2026.110859)
Supplement: 1 [file NIHMS2177814-supplement-1.docx]

**Supplemental MATERIAL**

**Supplemental Methods**

*Animals*

Male and Female C57BL/6J mice (n=124; The Jackson Laboratory; Bar Harbor, ME) were delivered at seven weeks of age, acclimated in standard group housing for one week, and then singly housed and acclimated for one week before stress exposure. All mice were maintained on a 12h light/dark cycle (lights on at 0600 hours) under controlled temperature (20-26°C) and humidity (30-70%) levels. Mice were given access to food and water *ad libitum*. All experimental procedures were conducted with the approval of the Institutional Animal Care and Use Committee at Wake Forest University and were within the guidelines set forth by the Care and Use of Mammals in Neuroscience and Behavioral Research (2003).

*Stereotaxic Surgeries*

Mice (>8 weeks old) were anesthetized with isoflurane (3% initial dose, 1.5% maintenance dose) for intracranial recombinant adenosine-associated virus (AAV) injection and fiberoptic cannula implant surgeries using a Leica Angle Two Small Animal Stereotaxic Instrument. All viruses AAV1-hSyn-jGCaMP8f-WPRE (Addgene #162376, titer ≥ 7×10¹² vg/mL), AAV5-hSyn-DIO-hM4D(Gi) (Addgene #44362, titer ≥ 7×10¹² vg/mL), and AAVrg-hSyn-Cre-WPRE-hGH (Addgene #105553, titer ≥ 7×10¹² vg/mL) were used as received. Mice were injected with 300 nL of the indicated AAV at a rate of 50 nL/min driven by a Micro4 MicroSyringe pump (World Precision Instruments) into the specified region. The needle (World Precision Instruments Nanofil syringe, fitted with a Nanofil 33G Blunt Needle) remained in place for an additional 5 minutes. A minimum period of 4 weeks (6 weeks for retrograde Cre-recombinase) was allowed before further experimentation to ensure full viral expression and recovery. Injection and implantation sites included the mid-insula (from Bregma: AP = 0.02, ML ± 3.66, DV = −4.30, 0° angle) and dorsal BNST (from Bregma: AP =0.20, right ML -0.63, left ML -2.13, DV = −3.88, 15.0° angle). Coordinates were obtained using a flat skull when bregma and lambda were within 0.05 cm D/V. The DV is taken from the skull surface in relation to bregma (Cecyn and Abrahao, 2023). All mice received 5 mg/kg injections of meloxicam once a day for 2 days following surgery. For chemogenetic hM4Di studies, the DREADD agonist C21 (MilliporeSigma) was diluted in saline and administered (1 mg/kg, i.p.) 1 hour before stress; control groups either received c21 injections without the hM4Di virus or received vehicle (saline) injections with the hM4Di virus. After confirming no behavioral differences between these two control groups, they were combined to increase statistical power.

Chronic optical fiber implantation procedures.

Once exposed, the skull was etched with a gel etchant (Kerr Dental). One stainless-steel mounting screw (PlasticsOne) was installed in the ipsilateral parietal plate posterior to the implant hole. Once the implant was inserted into the brain, it was bonded using Optibond primer, followed by Optibond adhesive, and then cured with UV light. Herculite enamel was molded around the screw, implant, and skull. The enamel was then cured with UV light. Mono-fiberoptic cannulas (Doric Lenses) were tested before implantation to ensure a power output of greater than 90%.

*Restraint Stress*

Restraint stress was conducted as previously described (Luchsinger et al., 2021). In brief, following a one-hour acclimation period and, when applicable, 30 minutes after drug administration, mice were placed in a custom restraining device, RESTRAINT (Luchsinger et al., 2021; Williford et al., 2023), for one hour. Videos were recorded using a custom data acquisition pipeline (Centanni and Smith, 2023) and subsequently processed through the DeepLabCut pose estimation pipeline (Mathis et al., 2018). A custom R script was used to determine the independent velocity of body movement while the subject was in the tube. A whole-body struggle bout was quantified as simultaneous movement of the head and tail tip.

*Chronic Drinking Forced Abstinence Model*

Chronic drinking followed by forced abstinence was done as described previously (Centanni et al., 2019). Briefly, beginning at 7–8 weeks of age, mice were single-housed and given access to two sipper bottles filled with water. After 1 week of habituation, our experimental group was given access to increasing ethanol concentrations (3% for 3 days, 7% for 7 days) until reaching a maintenance concentration of 10% for the remaining 5 weeks. The control group continued to drink from two bottles, each containing water, throughout the experiment. After 6 weeks of ethanol or water, ethanol was removed, commencing a forced abstinence period where behavioral testing took place.

*Abstinence Behavioral Tests*

Behavioral assessments of affective state were conducted as follows:

Novelty Suppressed Feeding Test (NSFT)

NSFT was conducted as previously described(Centanni et al., 2019; Holleran et al., 2016; Pang et al., 2013; Vranjkovic et al., 2018). In brief, 13 days into abstinence, mice were food-restricted for the 48 hours preceding the test. Food access was granted for 2 hours, 23-25 hours before NSFT. On day 15 of abstinence, mice were subjected to NSFT. Mice were placed into an open arena (50cm x 50cm) with 2 inches of home cage bedding, and a food pellet in the center of the brightly lit apparatus (300 lux). Latency to eat was measured as the amount of time elapsed before the subject took a bite of the food pellet. The latency to eat was assessed in real time by the experimenter and confirmed through video recording after the experiment. Mice were removed immediately after the first bite or if they did not take a bite within 15 minutes of the test beginning. Mice were then returned to their home cage with a pre-weighed food pellet. After 10 minutes, the food pellet was reweighed to determine the amount consumed in the home cage. During subsequent data analysis in time-locking interactions with the food, in interaction was defined as when the mouse moved to at least 2 cm and the time-stamp for when the mouse first enters that 2cm area was used to extract insula GCaMP activity (Fig. 3). The interaction time-stamps were calculated using custom R code together with pose estimation data from DeepLabCut.

Acoustic Startle Response

Testing was conducted in sound-attenuated startle chambers (SRLAB, San Diego Instruments, San Diego, CA), each consisting of a Plexiglas cylinder (3.8 cm diameter) mounted on a Plexiglas platform, with a high-frequency loudspeaker (28 cm above the cylinder) producing all acoustic stimuli. The background noise of each chamber was 70 dB. Movements within the cylinder were detected and transduced by a piezoelectric accelerometer, then digitized and stored by a computer. Three different acoustic startle levels were presented for each subject: startle pulse of 105 dB, 95 dB, or 90 dB, with three trials given for each startle level. Trials were given in random order. The average inter-trial interval was 67 seconds. Startle amplitudes were averaged across the different stimulus trials for each mouse before statistical analysis. Elevated startle amplitudes were interpreted as anxiety-like behavior.

Foot Shock Startle Response

Testing was conducted in the same chamber as previously described in the acoustic startle response. Footshock voltage levels were (mV) 0.05, 0.1, 0.2, 0.4, 0.6, and 0.8, with three trials for each shock level. Trials were randomized similarly to the acoustic startle.

*Fiber Photometry*

Data Acquisition: Fiber photometry was recorded using a TDT RZ10X fiber photometry system with Synapse acquisition software. Briefly, light from the 470 nm, 17.2mW (Min) fiber-coupled LED (Thorlabs) and light from the 405 nm, 19.3mW (Min) fiber-coupled LED (Thorlabs) were directed into a fluorescence mini cube with six ports and a built-in detector head (Doric Lenses), with spectral bandwidths of 405 and 470 nm. A 405 nm light was modulated at 217 Hz, while 470 nm light was modulated at 330 Hz. Power output was maintained at 20 mA with a DC offset of 3mA for both wavelengths. The light was then directed through a low-autofluorescence mono fiberoptic patch cord with a 400 μm core (Doric). This fiber was connected to the monofiberoptic cannulas that were implanted into the insula (4mm length, 400/430 μm I/OD, 0.66 NA, 2.5mm diameter). The power output at the fiber tip was 25–30 μW. Fluorescent emission from the tissue was collected through the same fiber and was detected using a femtowatt photoreceiver. Signal acquisition was 1 kHz and low-pass filtered at 6 Hz. The 405 nm excitation channel served as an isosbestic, calcium-independent control wavelength for GCaMP, allowing for bleaching and movement artifact corrections when directly fit to the calcium-dependent 470 nm channel. MATLAB scripts from TDT were used to fit the 405 nm signal to the 470 nm signal using linear regression.

Data Analysis: Change in GCaMP-mediated signal was calculated as

$\frac{\Delta F}{F}=\frac{change in 470 nm signal change in 405nm signal}{change in 405 nm induced signal}$ (1)

Time-locked Z scores were then calculated from GCaMP-mediated signals as:

$Z = \frac{\frac{\frac{\Delta F}{F}}{mean of \frac{\Delta F}{F}}}{standard deviation of \frac{\Delta F}{F}}$(2)

For behavioral time-locking, the Z-score was calculated from the median deltaF/F during a two-second window, -5 to -3 seconds before the onset of an event. The maximum peak was determined as the peak Z-score after the onset of an event, relative to the baseline.

Frequency and amplitude of GCaMP transients were calculated using a Z-score normalization across the whole trace. An event was identified as a statistical outlier above the baseline noise (Z=1.96, 95% confidence of isolating a statistical outlier, p<0.05). An event ending was calculated as the point at which signal fell below z<1.96.

*Statistics*

Correlation analysis was conducted using Pearson’s correlation coefficient reported as r with [95% confidence interval]. The p-value for the correlation analysis was determined, and the slope of the line was found to be significantly different from zero. NSFT analysis was conducted using a parametric two-tailed unpaired Student’s *t*-test. Data were analyzed using GraphPad Prism 10. The machine learning algorithm DeepLabCut was used in conjunction with custom-written R code to quantify struggle bout behaviors during restraint stress, and to time-lock mouse interaction time points during NSFT to assess insula GCaMP activity. For continuous outcomes (e.g., ethanol consumption) across multiple treatment/comparative groups, ANOVA was used, along with an appropriate method for multiple comparisons (e.g., Dunnett’s test) relevant to the hypotheses being tested. For simple two-group comparisons, Welch’s t-test was used.

Clustering analysis

Silhouette plots, which calculate the average distance between data points, were used to determine how well each data point fit into clusters. Hierarchical clustering plots and Gaussian Mixed Models (GMM) were used as further evidence determine the optimal number of clusters in the control conditions, which revealed a best fit of two clusters. K-means algorithms restricted k=2 were used to visualize multidimensional clustering. The robustness of the resulting model was validated through multivariate separation and sensitivity testing detailed in the tables below. Phenotypic segregation was quantified using Mahalanobis distance (De Maesschalck et al., 2000), and model stability was rigorously evaluated against potential sample size bias using stratified bootstrapping and balanced downsampling (C<3%). All analysis codes (clustering, photometry, behavior) were custom written by the authors using R-Studio and python. AI assistance (e.g., Microsoft Co-Pilot, Google Gemini) was used to troubleshoot, optimize, and confirm robustness and reliability of all codes.

**Supplemental Figures
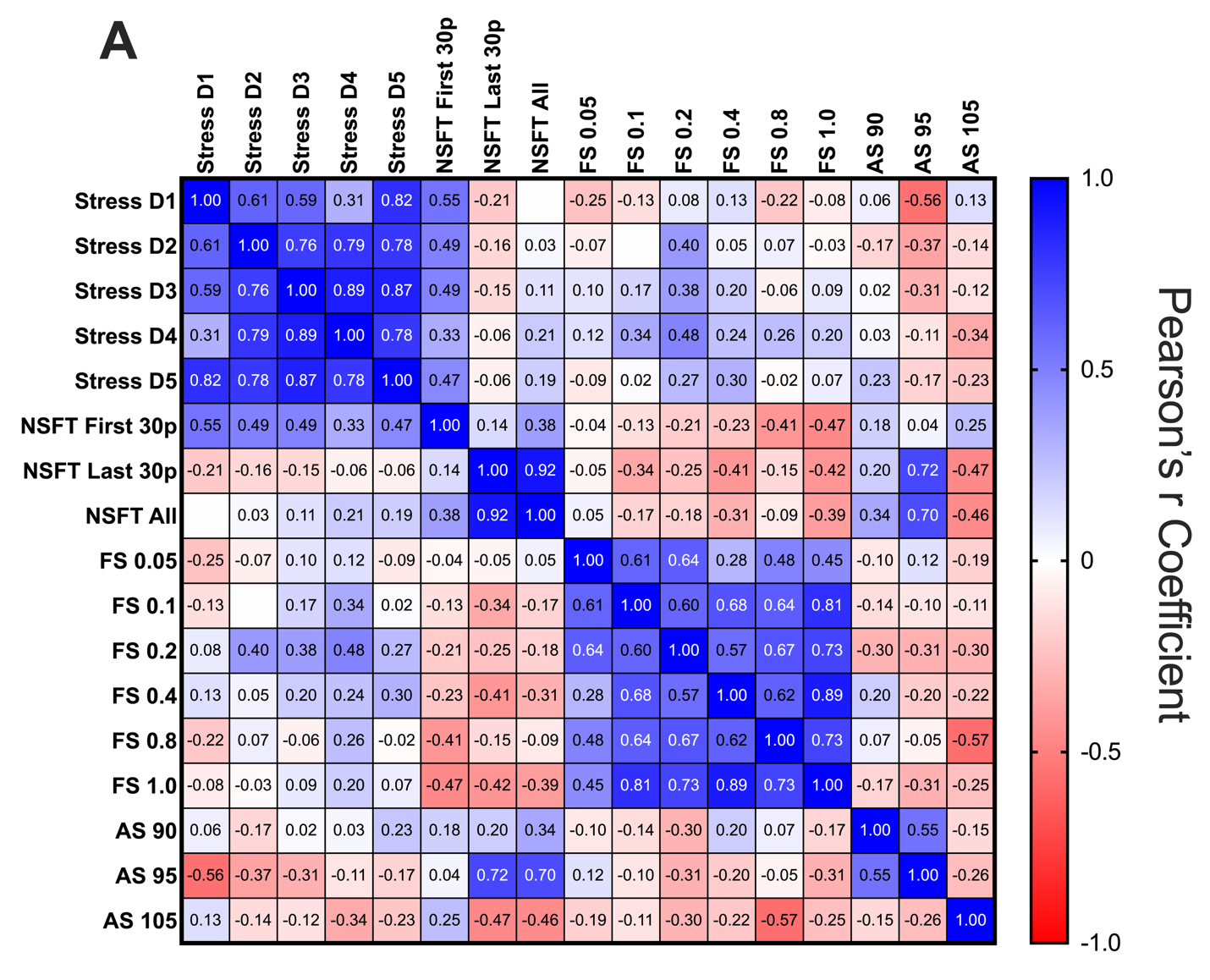
Supplemental Figure 1. The relationship between struggling behavior, mid-insula GCaMP spike activity during stress, and ethanol consumption in female mice.** (A) Correlation matrix displaying Pearson’s r Coefficients comparing struggling behaviors and mid-insula GCaMP spike activity both during and outside of struggle bouts during stress, and subsequent early vs late ethanol consumption. GCaMP spike during and outside of struggle bouts. AVG = average. D1-D5 = day1 through day 5.

**
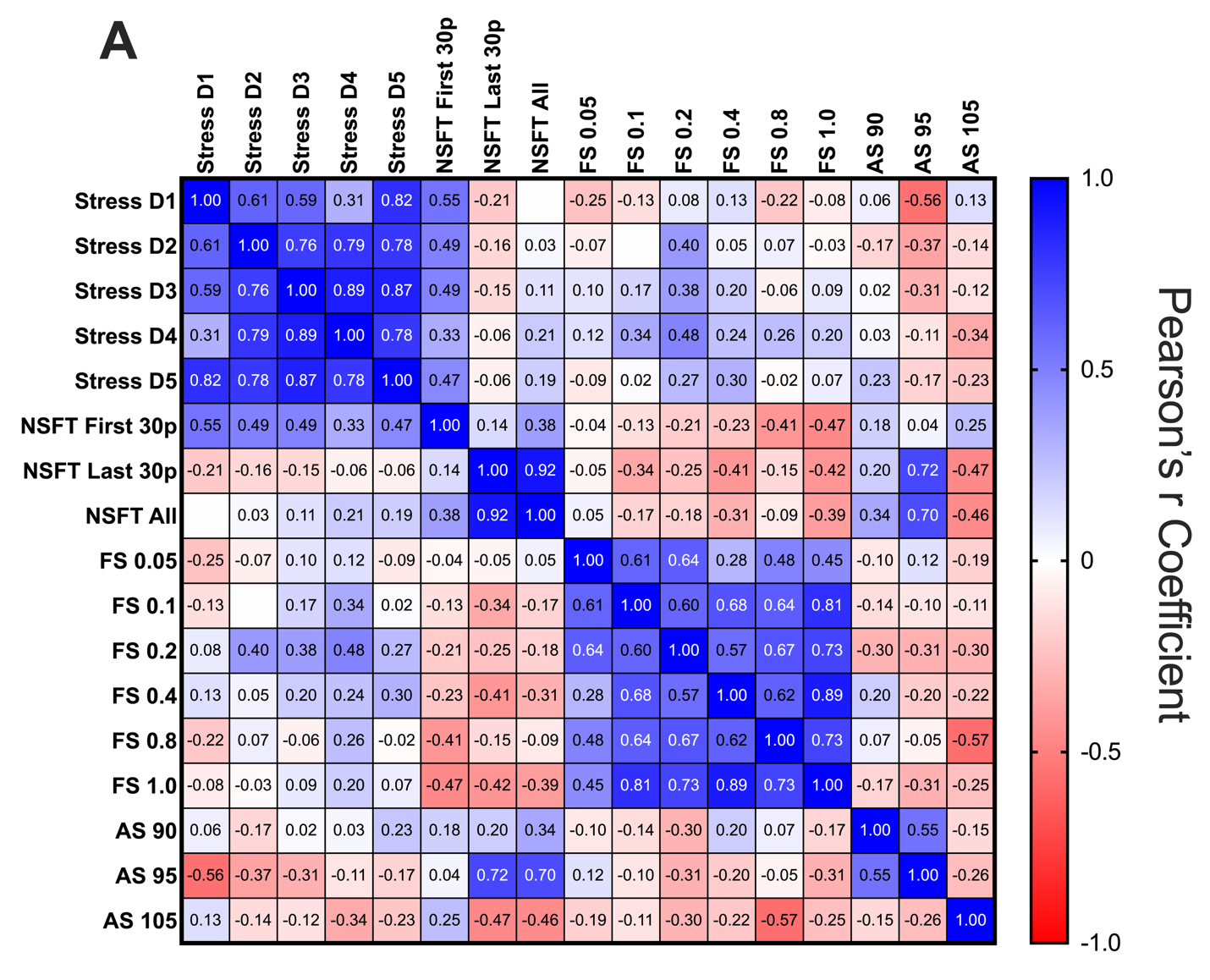
Supplemental Figure 2. The relationship between mid-insula GCaMP peak amplitude and time locked to struggle bout onset and aversive behaviors in abstinence.** (A) Correlation matrix displaying Pearson’s r Coefficients comparing GCaMP peak amplitudes time-locked to multiple behaviors after 2 weeks of abstinence from EtOH. Stress D1 – D5 = restraint stress days 1-5, NSFT First = first food interaction, NSFT Last = last consummatory food interaction, FS = footshock startle (values given in mV), AS = acoustic startle (values given in dB).**Supplemental Figure 3. Extended EtOH drinking data with drinking vs NSFT correlations.** (A-B) Male, but not female, hM4Di mice had lower drinking preference compared to stress controls. C-D). Food consumption during a 10-minute home cage consumption period after NSFT. C) Male control mice consumed a similar amount after NSFT compared to hM4Di mice. D) There were no differences in post-NSFT consumption in female mice. Stress and water group data are derived from Supplemental Figure 5 below. (E-F) Ethanol consumption negatively correlated with latency to eat during NSFT in males, but not in female mice. G-H) Food deprivation-induced weight loss before NSFT. G) Positive correlation between food deprivation-induced weight loss and latency in control mice (p=0.004) and a trending correlation in hM4Di male mice (p=0.078). H) There was no relationship between latency to eat and weight loss in female mice.


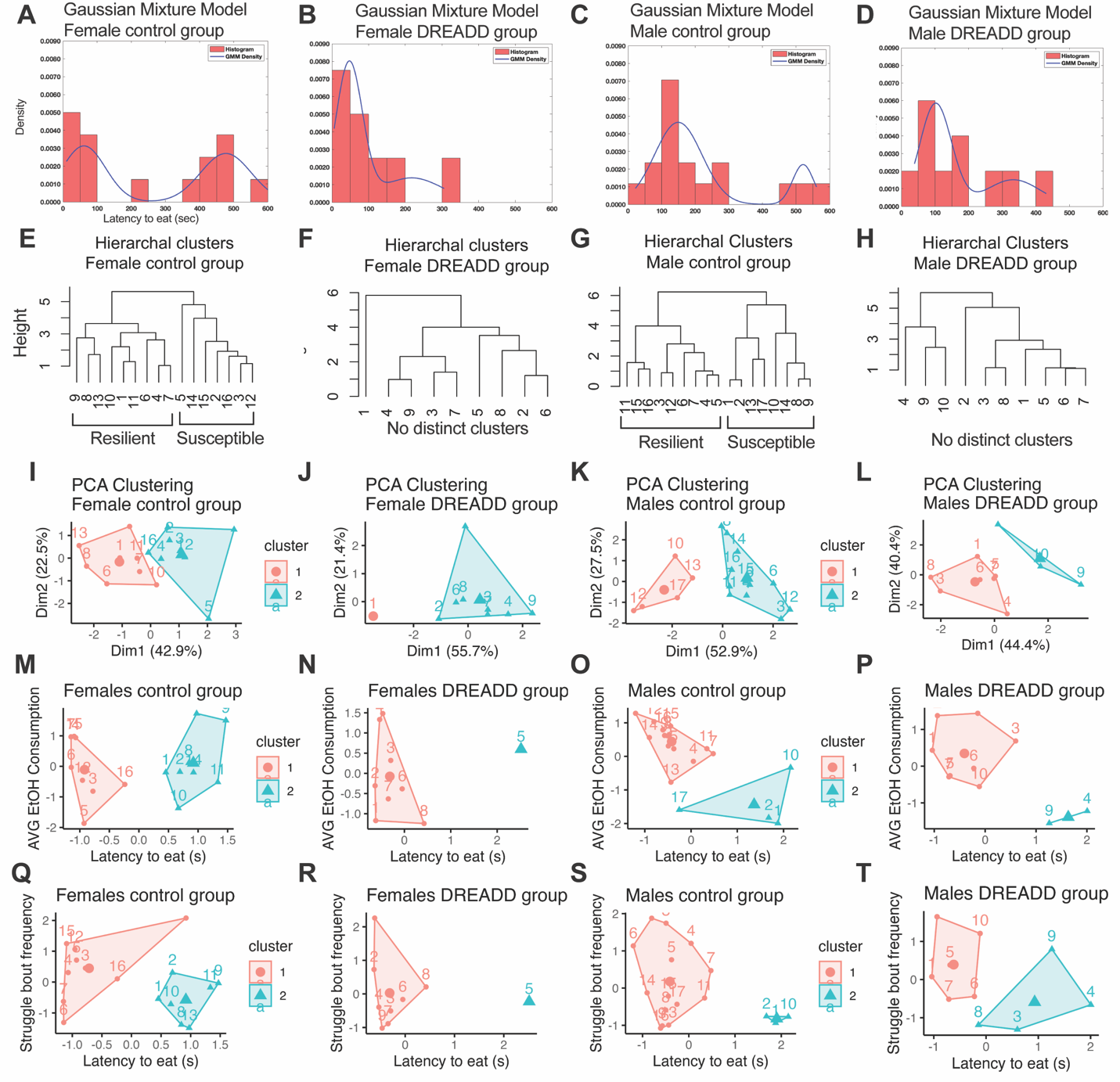


**Supplemental Figure 4. Clustering analysis of mouse behavior is affected by insula-BNST pathway inhibition during stress.** (A-D) Histogram of NSFT latencies binned into 50-second bins. Gaussian Mixed Model (red line) identifies modality distribution. (A, C). Female, and to a lesser extent, male control mice display a bimodal distribution, characterized by two distinct peaks. (B, D) hM4Di-C21 treatment during stress reveals a unimodal distribution in male and female mice. (E-H) Data exploration using hierarchical clustering to determine the optimal number of clusters in the data. (E, G) Hierarchical clustering suggests that the female and male control groups form two distinct clusters (F and H), whereas the female and male hM4Di DREADD groups are absent from these clusters. (I-L) Combining PCA dimensionality reduction with k-means clustering using the factoextra package in R to visualize two data clusters, including struggling behaviors during stress, ethanol consumption, and negative affective behavior during abstinence. (I, K) Female and male control mice appear to have two distinct data clusters, which are less well-defined in the female and male hM4Di DREADD groups (J, L). (M-P) Comparing two specific variables, average ethanol consumption and latency to eat during NSFT, and implementing the same clustering method as in I-L. (M, O) Male and female control mice exhibit distinct clusters, which are absent when forcing two clusters in the (N, P) female and male hM4Di DREADD groups. (Q-T) Comparing two specific variables, struggle bout frequency during restraint stress and latency to eat during NSFT, and implementing the same clustering method as in I-L. (Q, S) Female control mice exhibit two distinct clusters when comparing stress and NSFT; however, male control mice have less distinct clusters. (R) Female hM4Di DREADD mice do not have distinct clusters when comparing stress and NSFT (T), while male hM4Di DREADD mice do appear to have two clusters, unlike the female data.

**Supplemental Figure 5. Chemogenetic inhibition of the insula-BNST pathway does not affect negative affective behavior in ethanol-naïve mice.** (A) Experimental design. hM4Di was injected into insula-BNST neurons. C21 was administered before each stress exposure. Instead of CDFA, mice were left undisturbed for two weeks before NSFT testing. (B) No difference in latency to eat during NSFT between control and hM4Di groups.

**Supplemental Tables**

**Supplemental Table 1.** Stability and Sensitivity Analysis of female cluster phenotypes.

| **Analysis Phase** | **Metric** | **Observed Value** | **Confidence/Stability Metric** |
| --- | --- | --- | --- |
| **Model Selection** | Optimal Microclusters (k) | 2 | ARI=1.0 (100% Stability) via Bootstrapping |
| **Phenotypic Separation** | Mehalanobis Distance | 3.40 | Bootstrap 95%  CI: [2.85, 3.92] |
|  | Pooled Covariance (S) | 4.02 | Consistency across downsamples: CV=2.63% across downsamples |
| **Occupancy Analysis** | Control cluster 1 vs 2 | 8/8 | Balanced downsample: consistent |
|  | hM4Di cluster 1 vs 2 | 7/1 | Shift Stability: 92% |
| **Statistical Test** | Fisher’s Exact (p) | 0.22 | Downsampled p range [0.08-0.29] |
| **Model Fit** | Avg Silhouette Score | 0.37 | Median Bootstrap: 0.37 [IQR: 0.33-0.43] |

**Supplemental Table 2.** Stability and Sensitivity analysis of male cluster phenotypes.

| **Analysis Phase** | **Metric** | **Observed Value** | **Confidence/Stability Metric** |
| --- | --- | --- | --- |
| **Model Selection** | Optimal Microclusters (k) | 2 | ARI=1.0 (100% Stability) via Bootstrapping |
| **Phenotypic Separation** | Mehalanobis Distance | 3.91 | Bootstrap 95%  CI: [2.85, 3.92] |
|  | Pooled Covariance (S) | 4.59 | Consistency across downsamples CV=2.86% across downsamples |
| **Occupancy Analysis** | Control cluster 1 vs 2 | 9/7 | Balanced downsample: consistent |
|  | hM4Di cluster 1 vs 2 | 6/4 | Shift Stability: 92% |
| **Statistical Test** | Fisher’s Exact (p) | 0.70 | Downsampled p range [0.65, 0.97] |
| **Model Fit** | Avg Silhouette Score | 0.40 | Median Bootstrap: 0.40 (IQR:0.35-0.45) |

**Supplemental References**

2003. Guidelines for the Care and Use of Mammals in Neuroscience and Behavioral Research, Washington (DC).

Cecyn, M. N., Abrahao, K. P., 2023. Where do you measure the Bregma for rodent stereotaxic surgery? IBRO Neurosci Rep 15, 143-148.

Centanni, S. W., Morris, B. D., Luchsinger, J. R., Bedse, G., Fetterly, T. L., Patel, S., Winder, D. G., 2019. Endocannabinoid control of the insular-bed nucleus of the stria terminalis circuit regulates negative affective behavior associated with alcohol abstinence. Neuropsychopharmacology 44, 526-537.

Centanni, S. W., Smith, A. C. W., 2023. PiRATeMC: A highly flexible, scalable, and low-cost system for obtaining high quality video recordings for behavioral neuroscience. Addict Neurosci 8.

De Maesschalck, R., Jouan-Rimbaud, D., Massart, D. L., 2000. The Mahalanobis distance. Chemometrics and Intelligent Laboratory Systems 50, 1-18.

Holleran, K. M., Wilson, H. H., Fetterly, T. L., Bluett, R. J., Centanni, S. W., Gilfarb, R. A., Rocco, L. E., Patel, S., Winder, D. G., 2016. Ketamine and MAG Lipase Inhibitor-Dependent Reversal of Evolving Depressive-Like Behavior During Forced Abstinence From Alcohol Drinking. Neuropsychopharmacology.

Luchsinger, J. R., Fetterly, T. L., Williford, K. M., Salimando, G. J., Doyle, M. A., Maldonado, J., Simerly, R. B., Winder, D. G., Centanni, S. W., 2021. Delineation of an insula-BNST circuit engaged by struggling behavior that regulates avoidance in mice. Nat Commun 12, 3561.

Mathis, A., Mamidanna, P., Cury, K. M., Abe, T., Murthy, V. N., Mathis, M. W., Bethge, M., 2018. DeepLabCut: markerless pose estimation of user-defined body parts with deep learning. Nat Neurosci 21, 1281-1289.

Pang, T. Y., Renoir, T., Du, X., Lawrence, A. J., Hannan, A. J., 2013. Depression-related behaviours displayed by female C57BL/6J mice during abstinence from chronic ethanol consumption are rescued by wheel-running. Eur J Neurosci 37, 1803-1810.

Vranjkovic, O., Winkler, G., Winder, D. G., 2018. Ketamine administration during a critical period after forced ethanol abstinence inhibits the development of time-dependent affective disturbances. Neuropsychopharmacology.

Williford, K. M., Taylor, A., Melchior, J. R., Yoon, H. J., Sale, E., Negasi, M. D., Adank, D. N., Brown, J. A., Bedenbaugh, M. N., Luchsinger, J. R., Centanni, S. W., Patel, S., Calipari, E. S., Simerly, R. B., Winder, D. G., 2023. BNST PKCdelta neurons are activated by specific aversive conditions to promote anxiety-like behavior. Neuropsychopharmacology.
